# Optical sensors for determination of biogenic amines in food

**DOI:** 10.1007/s00216-020-02675-9

**Published:** 2020-05-08

**Authors:** Alexandra I. Danchuk, Nadezhda S. Komova, Sarah N. Mobarez, Sergey Yu. Doronin, Natalia A. Burmistrova, Alexey V. Markin, Axel Duerkop

**Affiliations:** 1 Institute of Analytical Chemistry, Chemo and Biosensors, University of Regensburg, 93040 Regensburg, Germany; 2 Institute of Chemistry, Saratov State University, Saratov, Russian Federation 410012

**Keywords:** Optical sensor, Biogenic amine, Colorimetry, Fluorescence, SERS, Dipstick, Food quality, Reflectometry, Chemiluminescence, Ellipsometry

## Abstract

This review presents the state-of-the-art of optical sensors for determination of biogenic amines (BAs) in food by publications covering about the last 10 years. Interest in the development of rapid and preferably on-site methods for quantification of BAs is based on their important role in implementation and regulation of various physiological processes. At the same time, BAs can develop in different kinds of food by fermentation processes or microbial activity or arise due to contamination, which induces toxicological risks and food poisoning and causes serious health issues. Therefore, various optical chemosensor systems have been devised that are easy to assemble and fast responding and low-cost analytical tools. If amenable to on-site analysis, they are an attractive alternative to existing instrumental analytical methods used for BA determination in food. Hence, also portable sensor systems or dipstick sensors are described based on various probes that typically enable signal readouts such as photometry, reflectometry, luminescence, surface-enhanced Raman spectroscopy, or ellipsometry. The quantification of BAs in real food samples and the design of the sensors are highlighted and the analytical figures of merit are compared. Future instrumental trends for BA sensing point to the use of cell phone–based fully automated optical evaluation and devices that could even comprise microfluidic micro total analysis systems.

## Introduction

**Fig. 1:**
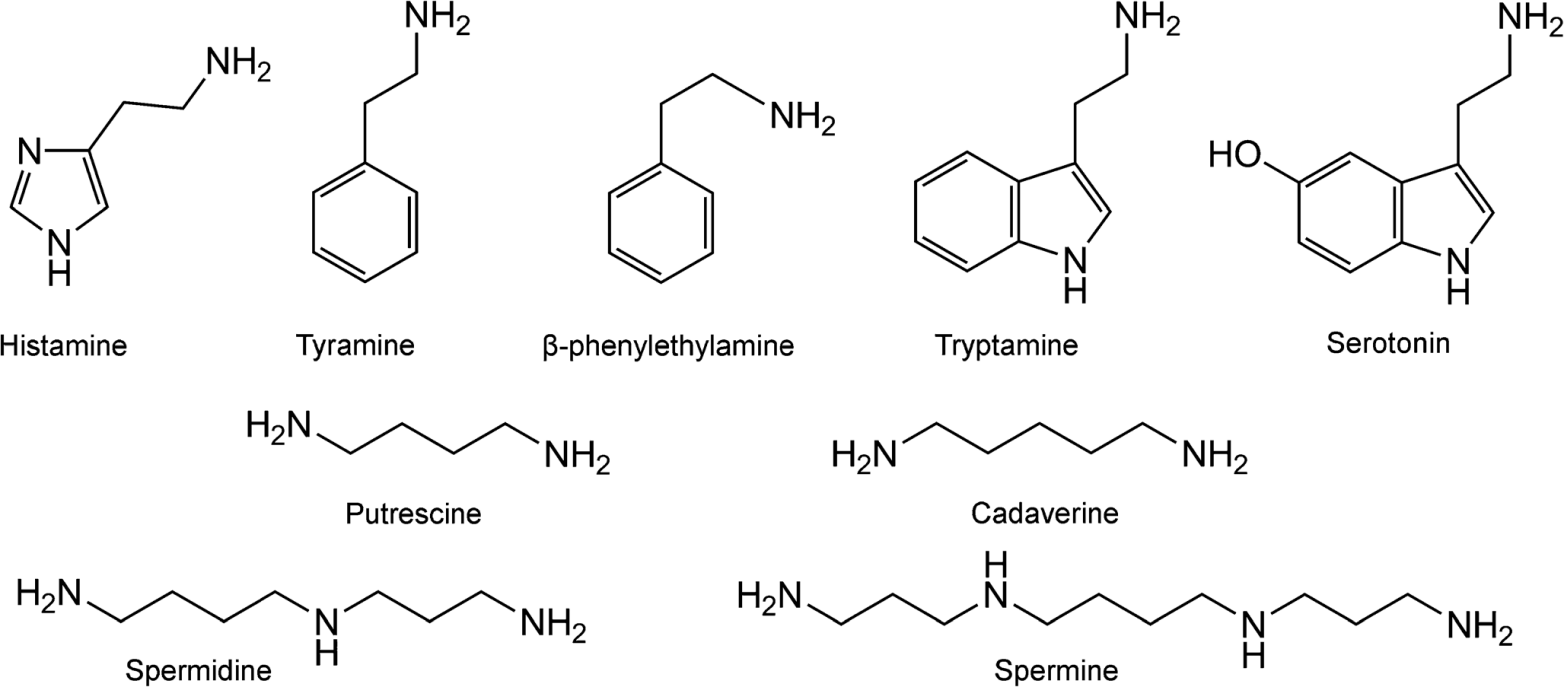
Molecular structures of main biogenic amines present in food

Biogenic amines (BAs) are small organic molecules, which show high biological activity. They mainly arise in tissues of living organisms as a result of enzymatic decarboxylation of amino acids or by amination and transamination of aldehydes and ketones. In fresh foods, they are mostly found in protein-rich samples but their concentrations in any food can quickly increase upon improper storage. The molecular structure of some main BAs which can occur in food and which are involved in food poisoning is shown in Fig. 1.

This review focuses in detail on optical chemosensor systems for BA determination in quality control of real food samples and particularly highlights the analytical aspects of the various optical detection methods. Commonly, different types of probes (organic dyes, metal-ligand complexes, nanomaterials, enzymes, etc.) and sensor designs as well as signal readouts find their way into BA sensors, and hence, those are classified here with respect to the various optical detection methods.

## Biogenic amines as indicators of food spoilage

Fish, meat, cheese, vegetables, fruits, nuts, and chocolate and various beverages like wine or beer are typical foodstuffs in which BAs are present at various concentration levels. The concentration of BAs in food depends on their nature and bacterial environment. Although BAs can act as hormones and neurotransmitters, their potential toxicity accounts for their significant role in food analysis [1, 2]. Papageorgiou and co-workers summarized different matrices (food and beverage products) in their review [2] that could contain biogenic amines in order to show which of them should be continuosly monitored for their toxicity. Moreover, the regulation policy and the toxicity of BAs are discussed.

Histamine is one of the most bioactive and toxic BAs, which can trigger an allergic reaction in humans and mammals. According to the European Food Safety Authority (EFSA), the U.S. Food and Drug Administration (FDA), and the World Health Organization (WHO), there are some ranges of BA concentration in foodstuff which point to various levels of food quality or food degradation. A concentration of histamine of less than 50 mg/kg indicates good-quality fresh food. Concentrations of histamine between 50 and 200 mg/kg may cause adverse health effects and levels above 200 mg/kg of histamine are reported to cause toxic effects in humans. Cadaverine and putrescine can potentiate the toxic effect of histamine. Poisoning of BAs on the one hand depends on the amount that has entered the body and on the other hand also on the individual human sensitivity to each BA. The permissible concentrations of other BAs are greater than that of histamine. Therefore, e.g., doses of tyramine can vary from 600 up to 2000 mg, depending on the individual tolerance of each person [2]. Inappropriate storage temperature often is the reason for high concentrations of histamine in fish which have formed following decarboxylation under bacterial action from initial histidine [3]. Tyramine and β-phenylethylamine may initiate a hypertensive crisis in certain patients and dietary-induced migraine [4]. Polyamines (putrescine, spermine, spermidine, and cadaverine), however, play essential functions in living cells and can be present in micro- or even millimolar concentrations. On the other hand, some polyamines can react with nitrite to form carcinogenic nitrosamines, and can also be food spoilage indicators [4]. Therefore, monitoring of BAs is a mandatory part of food analysis as a quality indicator for the sample under investigation.

BA detection in food is challenging because these analytes are very polar and better soluble in water than in organic solvents (Table 1, left column). Furthermore, BAs need to be detected at low concentrations in a complex matrix, in most cases. Hence, sensitive detection methods are required. Furthermore, high selectivity should be provided to avoid that interfering components in food may give a similar response as the target BA. The presence of structurally similar BAs in the sample can further complicate the analysis as a result of competitive interactions with the sensing material [2]. Therefore, the application of a new sensor to a specific food sample should be validated by a chromatographic method to warrant sufficient selectivity. BA detection by optical analytical methods is often complicated because of the weak capability of BAs to absorb light in the visible range. Therefore, derivatization of BAs with chromophores or fluorophores is employed to make them amenable for being optically analyzed. Further, a huge number of separation methods like GC, HPLC, or capillary electrophoresis is hyphenated with optical, electrochemical, or mass spectrometric detection as shown in recent reviews [4–8]. Finally, ELISAs [9] are applied for BA determination in foodstuff, as well. The main advantages of those methods are their high sensitivity and selectivity. However, they require highly qualified and trained staff, expensive high-quality reagents, and time-consuming sample preparation (Table 1, left column). In order to overcome these limitations, the recent development of fast, low-cost, and portable chemo and biosensors which can be used for on-site analysis of BAs in food is shown by recent publications covering about the last 10 years, in this review. Kaur et al. [10] published a comprehensive review describing the existing probes and their recognition mechanisms (including, e.g., aggregation-induced emission (AIE), ligand exchange mechanism, photo-induced electron transfer (PET)/internal charge transfer (ICT) mechanism, and some others) used for detection of biothiols and biogenic amines. Unlike this earlier publication, the present one focuses on evaluating whole sensors (comprising of probes, transducer, and detector), discussing on-site use capability, and comparing the merits and disadvantages of the various optical detection methods together with discussing the impact of sensor material design (in view of the materials used for embedding of the probe and exclusion of unwanted interferents) on sensor performance. From this wealth of perspectives on the topic, it is obvious that optical sensors for qualitative and quantitative analysis of BAs are an attractive alternative to existing analytical methods and some of them may be handled by non-trained staff or in-field.

**Table 1:** Challenges to be solved in the determination of BAs in real food samples and merits of optical sensors that promote their use

Challenges of BA determination	Advantages of optical sensors
a. derivatization of BAs and compliance with green chemistry; b. strong polar character of BAs; c. low concentration ranges of BAs; d. presence of complex matrix with potentially interfering compounds; e. occurrence of several BAs simultaneously; f. complexity of sample matrix; g. time for sampling, work-up, and detection; h. requirement of trained personnel.	General: a. fast signal reading and processing; b. low-cost instrumentation; c. minimum amount of sample required; d. ease of operation; e. evaluation by public domain software; f. acceptable for analysis by less-trained users; g. flexible size from μm^2^ to cm^2^; h. multiplexing/array sensing possible; i. amenable to remote sensing; j. no electrodes required. Luminescence sensors: general advantages as from a–j; k. high sensitivity. Surface-enhanced Raman spectroscopy sensor: general advantages as from a, c, d, f–j; l. high sensitivity.

## Sensor design and detection methods

Most of the optical sensors for BAs follow the classical sensor design where a probe for recognition is immobilized in a membrane and/or fixed on a suitable support which can be paper [11–13], glass [12, 14], TLC plates [15–17], microtiter plates [18], and test tubes [19]. To deposit the recognition layer, knife coating, spin coating, or simple soaking may be used. The probe is mostly embedded into a polymer layer as a thin film [15, 20], or eventually allowed to settle on another film of, e.g., gold or silicon [21], on polymer particles [18, 22, 23], on a hydrogel [24], or inside nanofibers [25]. Then, various optical methods are applied for detection. Those will be discussed in the following sections together the individual sensor schemes and with their major merits towards quantitation of BAs in real samples. Only if these methods have shown to work in real food samples, they are presented in Table 2 together with an in-depth overview on sensor composition and individual sensor details like response times, analytical ranges, and limits of detection.

**Table 2:** Selection of representative sensors with proven applicability to real food samples and their analytical figures of merit

Composition of sensor layer/response time	Analyte	Detected signal/tool	Analytical range; LOD; (LOQ		BAs found in real sample, SD or RSD (%), *n* of samples	Object	Ref.
1	2	3	4		5	6	7
Reflectometric readout							
Cellulose filter paper; thiosemicarbazide–naphthalimide based chromophore/10 min	Butylamine	Reflectance/absorbance conversion/Ocean Optics spectrometer (Oceanview software)	3–224 mg/kg; –		–	Fish meat (salmon) (vapors)	[13]
Multi-dye (8 pH indicators and 8 porphyrins or metalloporphyrins) array printed on a TiO_2_ nanoporous film plate/9 min	Trimethylamine	RGB/Permeater flatbed scanner model PD-1B-2 (Gastec Corporation, Japan)	3.7–10.1 mg/kg*; –		Trimethylamine < 5 mg/kg SD = 0.05 mg/kg, *n* = 5	Yao-meat (vapors)	[15]
TLC plate with Meldrum’s acid–activated furan (MAF) as an amine-selective stain-based synthetic sequence (< 1 min) nylon filter membranes spotted with MAF/5 min	Dimethylamine	Visual detection, digital image/smartphone	0.3–2 mg/kg; 0.4 mg/kg		–	Fish meat (cod and tilapia) (vapors)	[16]
TLC plates pre-coated with silica gel G, diazonium reagent/15 min	Histamine	Optical density/mobile phone, ImageJ software	50–150 ng/spot 14.03 ppm; (39.15 ppm)		Histamine RSD = 1.07–2.76%, *n* = 6	Fish meat (mackerel) (vapors)	[17]
Array of pH indicators (methyl red, alizarin, bromophenol blue, thymol blue, chlorophenol red) cellulose acetate membranes/10 min	Isobutylamine, triethylamine, isopentylamine	RGB/iPhone®	2–8 ppm 2–10 ppm 1–4 ppm (1 mg/kg)		–	Minced meat (vapors)	[20]
Inkjet paper with monolayers array of hollow AuNPs received by reversal nanoimprint lithography/30 min	Putrescine	Shift of reflectance spectra	0.1–200 mg/kg; 13.8 mg/kg		–	Spiked fish meat (vapors)	[23]
Au NRs with Ag metallization/30 min	Trimethylamine	RGB/smartphone, visual detection	0.011–0.200 μM; 8.6 nM = 0.51 μg/kg *		–	Fish (salmon), beef meat (vapors)	[24]
Combination of 2-fluoro-4-[4-(2-hydroxyethanesulfonyl)-phenylazo]-6-methoxyphenol and Remazol Brilliant Blue R, immobilized on cellulose microparticles/1.5 h	Methylamine, dimethylamine, putrescine, cadaverine, histamine, tyramine and tryptamine	CIE lab color space/color measurement device (Mathi, Germany)	0.3–30 mg/kg –		–	Chicken, pork meat (micro-biological spoilage) (vapors)	[26]
Silica gel 60 F_254_ plates, 16 chemo-sensitive dyes/24 h to several days	Trimethylamine, dimethylamine, cadaverine, putrescine	Gray scale/Corel Photo- Paint X3, flatbed scanner (Epson V750-M Pro Perfection scanner)	81.7–350 mg/kg* –		Volatile basic nitrogen (TVB-N) 81.7 mg/kg* (1 day), 350 mg/kg* (9 day), RSD < 9%, *n* = 4	Fish meat (atlantic salmon) (vapors)	[27]
AuNPs (13.1 ± 0.7 nm)/15 min	Histamine	Color change/visual detection or camera; Absorbance/TU-1901 spectrophotometer	0.1–2.1 μM; 1.81 μM; 38 nM		Histamine 1.81 μM SD = 0.3–0.7 μM, *n* = 3	Fish meat (salmon) (solutions)	[28]
Photometric readout							
Polymeric film, nitrated conjugated polythiophene (NPTh)/2.5–37 min	Ethylenediamine, putrescine, cadaverine, spermidine, phenethylamine, histamine	Color change/visual detection or camera; Absorbance/UV–vis spectrophotometer (JASCO V-650)	– 5.6 mg/kg; 0.92 mg/kg; 0.45 mg/kg; – – –		–	Beef, pork, and salmon meat (vapors)	[29]
AuNPs (11–19 nm)	Histamine, histidine	Color change/eye-vision	2–16 μM; 0.6 μM		–	Poultry meat (solutions)	[30]
*o*-Phthalaldehyde (OPA) and thioglycolic acid (TGA) derivative of putrescine/12 min	Putrescine	Absorbance/UV–vis spectrophotometer (PerkinElmer Lambda 35, USA)	0.8–200 μM; 0.44 μM		Putrescine 41–180 mg/g RSD = 10–42%, *n* = 3	Fish meat (10 commercial products) (solutions)	[31]
Luminescence readout							
Cellulose acetate nanofibers embedded with Py-1/20 min	Tyramine	Fluorescence with RGB/digital camera Canon EOS 550D	10–100 μM; 20 μM		Histamine (μmol/g) 14.1 ± 0.3 (day 0), 16.4 ± 1.5 (day 3), 38.8 ± 2.6 (day 6) RSD = 17–21% *n* = 4	Shrimp meat (solutions; TAC)	[25]
Microtiter plate with sensor film based on Py-1 embedded in Hypan HN 80/10 min	Histamine, putrescine, tyramine	Fluorescence/FLUOstar Optima microtiter plate reader	Histamine: 0.5–70.0 mg/kg; 0.165 mg/kg; (0.495 mg/kg)		Total content of biogenic amines (TAC) 49.6–137.8 μg/mL (day 1), 97.6–397 μg/mL (day 5) SD = 0.23–2.19 μg/mL RSDs 3.71% (10.0 μg/mL), 4.08% (20.0 μg/mL), 3.73% (30.0 μg/mL) 5.03% (40.0 μg/mL) *n* = 5	10 samples of meat, cheese (solutions; TAC)	[32]
Tiss®-Link (NanoMyP®) support with immobilized tryptamine/15 min	Tryptamine	Phosphorescence/Varian Cary-Eclipse luminescence spectrophotometer with flow-through cell	19–600 μg/kg; 6 μg/kg; (19 μg/kg)		Tryptamine 366.8–697.6 ng/mL RSD = 1.8–6.1% *n* = 4	Beer (solutions)	[33]
DAO immobilized on chitosan-coated magnetic microparticles (MCH) or SEPABEADS® EC-HA (MFS) deposited with Ru-bathophenanthroline on a lens connected to optical fiber	Putrescine	Homemade time-correlated single photon counting instrument	MCH, mM 0.1–1.000; 0.025 = 2.2 mg/kg*; (0.082)	MFS, mM 0.1–0.750; 0.061 = 5.4 mg/kg*; (0.202)	–	Beer (solutions)	[34]
	Cadaverine		0.1–1.000; 0.030 = 3.1 mg/kg*; (0.102)	0.1–1.000; 0.052 = 5.1 mg/kg*; (0.175)			
	Spermidine		0.1–2.200; 0.271 = 39.6 mg/kg*; (0.905)	0.1–1.250; 0.093 = 13.5 mg/kg*; (0.309)			
	Histamine		0.1–2.200; 0.479 = 53.2 mg/kg*; (1.595)	0.1–1.250; 0.205 = 22.7 mg/kg*; (0.683)			
Chemiluminescence readout							
Glass support with hydroxyethyl cellulose membrane included luminol and covered with Co(II) and enzyme (putrescine oxidase or diamine oxidase) fixed on the photodiode	Putrescine	Chemiluminescence/Anthos Labtec Instruments Microplate luminometer Lucy1	Putrescine using putrescine oxidase: 1–2 mg/L; (0.8 mg/L); Putrescine using diamine oxidase: 1–2 mg/L; (1.3 mg/L)		Putrescine using putrescine oxidase 0.72–2.76 mg/L SD = 0.1–1 mg/L; Putrescine using diamine oxidase 0.78–2.45 mg/L SD = 0.3–1.1 mg/L *n* = 15	Beef, pork, chicken, turkey, and fish meat (solutions)	[18]
Bis(2,4,6-trichlorophenyl)oxalate(TCPO)–H_2_O_2_ system modified with Mg–Al–CO_3_–LDH nanosheet colloids	Histamine	Ingibition of chemiluminescence/Hitachi, F-7000 fluorescence spectrophotometer	0.1–100 μM; (3.2 nM = 0.4 μg/kg*)		Histamine 14.6–14.7 μM SD = 0.2–0.4 μM (day 12) 30.8–31.2 μM SD = 0.2–0.4 μM (day 5) *n* = 3	Fish, pork meat (solutions)	[35]
Surface-enhanced Raman spectroscopy							
AuNPs; preliminary solid phase extraction of the analyte by PVC film with incorporated MIP	Histamine	Raman spectrometer with 785-nm excitation wavelength	3–90 mg/kg –		–	Fish meat (canned tuna) (solutions)	[36]
AgNPs; preliminary liquid-liquid extraction of the analyte		Raman spectrometer with 514-nm excitation wavelength	10–200 mg/kg –		–	Fresh fish meat (solutions)	[37]
AgNPs; preliminary TLC separation of the analyte		Raman spectrometer with 633-nm excitation wavelength	15–100 mg/kg –		Histamine (1) 54.3 mg/kg, SD = 5.2 mg/kg RSD = 9.5% (2) 69.5 mg/kg SD = 6.8 mg/kg RSD = 9.8% *n* = 4	Fresh fish meat (solutions)	[38]
AgNPs; preliminary HPTLC separation of the analyte	Tyramine	Raman spectrometer with 633-nm excitation wavelength	30–80 mg/kg* –		Tyramine found 22.5–5.9 mg/kg SD = 0.7–2.1 RSD 6.7% *n* = 3	Cheese (solutions)	[39]

*Calculated by the authors of this review based on data from referenced manuscript

The optical BA recognition is correlated to binding to the receptor molecules that create the analytical signal. Unfortunately, many BAs themselves do not carry structural features that promote their optical detection (i.e., large conjugated π-systems which are necessary for reflectometric, photometric, or fluorimetric readout), so they need to be transformed into corresponding derivatives with the desirable detection properties [40]. The derivatization reagents may be divided into several groups: they can be chromophores and fluorophores (to impart absorption of UV light or fluoresce emission into the derivatives, respectively); fluorogenic molecules (which show fluoresce upon formation of the fluorescent derivative with the analyte); and redox reagents (which reduce/oxidize analytes to enable detection). For instance, reagents such as o-phthalic aldehyde, 1-fluoro-2,4-dinitrobenzene (DNFB), 2,4,6-trinitrobenzenesulfonic acid (TNBS), 4-fluoro-3-dinitro-fluoromethylbenzene, ninhydrin, benzoyl chloride, and many others were widely used for derivatization of primary or secondary amines or polyamines to form colored products, followed by their chromatographic determination with absorbance or a fluorescence detector. However, most of these methods require expensive instrumentation that cannot be used in the field and the related reagents cannot be used in optical sensors for BAs because they lack long-term storage stability inside the sensor membrane.

Optical sensors (so-called pH opt(r)odes) have the merit not to require electrodes and hence to be electrically safe which made them increasingly attractive in the fields of gas sensing, ion sensing, bioanalysis, and pH sensing. Optodes are based on changes of optical properties such as absorbance, fluorescence, luminescence, chemiluminescence, energy transfer, or reflectance by measuring the intensity of light in various regions of the spectrum (UV, visible, NIR, IR). Moreover, related properties such as light scattering, luminescence lifetime, refractive index (via surface plasmon resonance spectrometry), diffraction, and polarization may be exploited analytically. Optodes are beneficial due to their flexibility in size and shape, low cost, fast response, and light weight (Table 1, right column). They can be used in both aqueous and organic media and can deliver information with either highly local resolution (using fiber-optical sensors down to μm), which additionally can reduce the sample volume down to nanoliters. This is, e.g., hardly available for electrical sensors. Also areas until tens of square centimeters can be read using in optical imaging. A further benefit is the potential to use multiplexed sensing, e.g., when various optical probes with different detection wavelengths are used for simultaneously probing a collection of analytes or to build up and read out a sensor array [15, 20, 26]. Arrays of multiple sensors with similar and low selectivity often require multivariate data analysis methods to introduce analyte selectivity and also enable prediction of the freshness of food samples. Luminescence is the detection method of choice if high sensitivity is required because due to the absence of interfering excitation light, it is more sensitive than reflectometry and photometry. Sensors based on surface-enhanced Raman spectroscopy (SERS) present another modern highly sensitive alternative here because they are based on analyte-induced changes of the refractive index. For appropriate use in food sensing, these sensors may require either labeling or derivatization procedures in or on the sensor membrane or a sample pretreatment that involves analyte separation from the matrix to warrant selectivity. Leaching or photobleaching of the optical probe may compromise long-term stability and therefore has to be carefully controlled, as well as selectivity. Optical sensors are suitable for remote sensing (even over distances of kilometers) and can be used for in vivo measurements because of their immunity to electromagnetic interferences. This wide range of how optical sensors can be tailored makes them hot candidates for emerging into new fields like food chemistry that is dominated by more expensive separation methods (GC and HPLC) hyphenated to various optical, electrical, or mass detection techniques. Here, optical sensors can operate on a much less expensive level by using, e.g., digital cameras, smartphones, or flatbed scanners for detection together with public domain software for evaluation. The use of these inexpensive components provides a much easier operation of the sensors. This will further promote the use of optical sensors in food sensing as well as by less-trained personnel.

## Reflectometric readout

Frequently, colored or fluorescent dyes, such as acid-base indicators [15, 20, 26], porphyrins [14], phthalocyanines [21], chameleon dyes [11, 32], coumarine derivatives [12], azodyes [41], or nanomaterials, are used for optical BA determination. Among them, selectivity may be either introduced by a specific reaction, (co-)introduced by the material(s) the sensor is composed of, or is the result of chemometric evaluation of data from a sensor array. Such arrays may either be composed of several highly selective sensors to provide multi-analyte sensing or contain many sensors of low specificity [15, 20, 27] that may also be evaluated by pattern recognition. In general, reflectometric sensors require a light source, a wavelength-dispersive element to select the detection wavelength(s), and a detector. In modern setups, only one or few inexpensive LEDs (for array illumination) may be used as light source. Considering the narrow spectral bandwidth of those, selection of the light reflected from the sensor can be accomplished either by optical filters which are low-cost devices or by the detector itself if CCDs or CMOS sensors are used. These sensors typically deliver an RGB (red-green-blue) readout of a sensor array, as shown in several publications [15, 20, 25, 27] which can open a gate for multi-wavelength detection if the reflected light or different sensors matche the red, green, and blue spectral window of the array detector. The widespread use of those detectors in digital cameras and cell phones opens many new options for the use of these inexpensive devices for the readout of reflectometric sensors in food analysis and point-of-care diagnostics. The rapid sensor readout and simple evaluation by public domain or OEM software are further advantages of the reflectometric readout. Hence, the major advantages of reflectometric optical sensors are their instrumental simplicity, the option to arrange (multi-)sensor arrays with reasonable demand, and rapid evaluation with commercial detection equipment. Hence, a complete sensor (array) can be produced for a few hundred US $ or less. The major drawback of reflectometric sensors is their comparatively low sensitivity and accurate positioning of the sensor (spot) with respect to light source and detector is required.

In one study, an array of five pH indicators (methyl red, alizarin, bromophenol blue, thymol blue, chlorophenol red) in cellulose acetate membranes with Tween as plasticizer [20] was read in short time (10 min) to discriminate between isobutylamine, triethylamine, and isopentylamine in ppm concentrations through RGB readout via a cell phone. The mechanism of the color change is based on BA solubility and acidity constants. RGB analysis data of the color difference map of the membranes prior and after reaction with BAs was fed into chemometric analysis (principal component analysis and hierarchical cluster analysis) for non-supervised pattern recognition evaluation. This can help the readout of the array for users that are color blind because the three BAs could then be discriminated without misclassification. The membranes could be reused up to 14 times and were shown to work with meat samples.

In another sensor array, eight pH indicators (gentian violet, leucomalachite green, thymol, methyl yellow, bromophenol blue, Congo red, methyl orange, methyl orange) were used to detect trimethylamine in meat in the range of 60 ppb–10 ppm [15]. TiO_2_ nanoporous films were found to be superior as a support compared with C_2_ reverse silica gel plates on which colorimetric arrays of dyes were printed using microcapillary pipettes. Chemometric analysis was again required after trimethylamine (TMA) vapor was detected using reflectometric RGB color difference maps recorded by a flatbed scanner. The data from the color difference maps was analyzed with principal component analysis and multiple regression analysis and the use of partial least square models allowed the prediction of the TMA content in meat samples and their freshness. The nanoporous structure significantly improved homogeneity, sensitivity, and stability of the sensor arrays and lowered the response time minimally (9 min). A colorimetric sensor array for fish spoilage monitoring was evaluated [27] which included sixteen chemosensing compounds incorporated to silica gel 60 F_254_ plates using a micromilled polymethyl metacrylate mask. The sensor response was evaluated based on the overall contribution of all compounds estimated as color change of each spot before and after the exposure to the analyte. It was shown that the arrays including bromophenol blue, cresol red, and bromocresol green (group 3) and methyl red, xylenol blue, and crystal violet lactone (group 4) provided the highest response during fish (salmon) aging at r.t. and 4 °C. These arrays can be used as non-invasive, low-cost devices for monitoring food spoilage over time.

Cellulose-based microparticles were covalently conjugated with a pH indicator [26] and a blue reference dye and embedded into silicone. This yields slow (1.5 h) traffic light color changes of the sensor layer. The responses to BAs were reflectometrically acquired by a low-cost digital camera and evaluated in the lab color space of the CIE system for quantification of various amines and ammonia developed upon food aging in food packages. Paper-based plasmonic reflectometric sensors were fabricated using reversal nanoimprint lithography [23], which is a robust and rapid method for embedding of metal NPs in a substrate and subsequent transfer onto paper. Gas-phase analysis of BAs was carried out using a digital camera. Six types of paper were tested and a commercial inkjet paper showed the best result to sense putrescine in spiked fish at ppm levels in 30-min time.

Activated furans (MAF and BAF) were investigated as versatile probes for selective detection of amines in solution, on TLC sheets, and in the vapor phase [16] yielding a donor–acceptor Stenhouse adduct. Primary and secondary amines form a pink-colored (532 nm) product with MAF at a different rate which allows for their selective discrimination on solid-phase resins for peptide synthesis. Further, dipsticks for BA vapor detection from various fish samples were created by deposition of MAF on nylon membranes that were read with a smartphone and free software.

In other publication [17], histamine recognition in mackerel samples was carried out by visualization with ninhydrin and diazonium reagents with high specificity and sensitivity of latter for histamine using TLC plates (LOD 14 ppm).

A thiosemicarbazide–naphthalimide-based chromophore was also investigated [13] as a highly sensitive probe for visual and colorimetric determination of different amines including BAs with a color change from yellow to blue. The advantage of such a sensor is the regeneration capability of the chromophore (by trifluoroacetic acid) and hence the potential for a repeated colorimetric response. It was determined that the deprotonation and protonation processes can be repeated for at least six cycles.

Moreover, a sensor film containing a pyrylium salt (2,6-diphenyl-4(p-methacryloyloxy)-phenylpyryliumtetrafluoroborate) incorporated into a methacrylic polymeric membrane could be reused in presence of HCl vapors for at least 10 times. It selectively recognized trimethylamine with changing its color from yellow to pink [42] and LODs of 3.37 ppm and 4.42 ppm were found for colorimetric and photometric readouts, respectively.

Inorganic nanoparticles (NPs) have been widely used in optical sensing due to their stability, good solubility, and favorable luminescence properties [10, 43]. The aggregation-induced change of the surface plasmon resonance of stabilized gold nanoparticles (AuNPs) after interaction with histamine allows to create a colorimetric approach for on-site monitoring (LOD 38 nM) based on the electrostatic interaction of citrate-modified AuNPs with the ammonium group of the BAs [28]. Histamine was also detected visually down to 1.81 μM in fish samples from a red-to-blue color change. Similarly, diamine recognition of AuNPs modified by cucurbiturils or cyclodextrins was used for visual and spectrophotometric detection of cadaverine in aqueous solutions (LOD 3.9 μM) [22]. The aggregation-based assay has good selectivity but requires up to 150-min incubation time for high reproducibility. Au nanorods (obtained by a seed-mediated growth method) [24] were allowed to perform a hydrolysis-induced silver metallization that was used for colorimetric detection of BAs (LOD 8.6 nM) by the multiple color change and blue shift of the localized surface plasmon resonance (LSPR) of the gold nanorods.

## Photometric readout

Photometric sensing schemes have the merit that they are available in most labs, are essentially not more complex than reflectometric setups, and can be easily operated with little training. They share most of the advantages of reflectometry such as the requirement of only one or few (for reading arrays) inexpensive and simple light sources. Devices for the selection of the detection wavelength (e.g., filters) are only required if broadband light sources or multi-wavelength arrays are used. Hence, photometry can even be more simple than reflectometry. Similarly to the reflectometric readout, detection may be done with photodiodes, CCDs, or CMOS sensors. The latter two became more and more popular with the common use of digital cameras and cell phones. Those are most frequently employed, if sensor arrays are to be read out. The sensitivity of photometry is modest alike reflectometry, and for lower analyte concentrations, luminescence sensing is recommended.

In an alternative strategy to the reflectometric readout, e.g., the spectral shift of the absorption maximum of porphyrins or metalloporphyrins is used. Those probes are spun as films on glass slides and respond photometrically to volatile amines in the ppm range within 1 min (*t*_50_) at around 440 nm [14]. If deposited on a flexible transparent support, a use in food packaging seems feasible. Another team used AuNPs for detection of histamine (LOD 0.6 μM) as a biomarker for poultry meat freshness [30] either by photometry or by luminescence.

A nitrated conjugated polymer (NPTh) was employed for the photometric sensing of amines [29]. The polymer is a functional active material for optoelectronic devices and chemical sensors. It was synthesized from parent polythiophene and adsorbed into silica gel and its response was compared with non-BAs and non-amine compounds. BAs with a lone electron pair on the central nitrogen have electron-donating properties in contrast to NPTh because of the powerful electron-withdrawing character of its nitro group. After exposing of the polymer film to BA vapors (ethylenediamine, putrescine, cadaverine, spermidine, phenethylamine, and histamine), its color darkens and this induces a broad absorption band in a wide wavelength range of 300–700 nm. A wavelength of 450 nm shows the most pronounced response to BAs.

Determination of primary BAs (isopentylamine, propylamine, and putrescine) with the indicator dye ETH4001 immobilized in ormosil sensor layers formed by sol-gel technology was performed [44]. The sensing layers were read continuously by photometry at 520 nm in a flow-through cell and showed good reproducibility and high efficiency at millimolar concentrations with 5–15-min response times. The sensor foils were resistant against photobleaching and long-term stable for 9 months. This sensor has a good continuous on-site monitoring capability because it was used with appropriate miniaturized instrumentation.

A more long-wave photometric detection wavelength is achieved by the nucleophilic attack of 1-propylamine at an azo dye to irreversibly convert its tricyanovinyl group into a 1-propylamino-2,2-dicyanovinyl group. A sensor membrane with this dye embedded in plasticized poly (vinylchloride) on glass responded to BAs after modest 15–30 min with an absorbance decrease at 630 nm in a homemade flow-through cell in a UV–VIS spectrometer [41]. The response of the sensor is less linear at higher concentrations and the sensor layer is stable up 6 months. The linear quantitation range is from hundreds of micromolars to 4.0 mM of 1-propylamine.

A coumarin derivative yields covalent enamine adducts with BAs and enables not only ratiometric photometric (377/403 nm) but also fluorescence sensing (470 nm) [12] in the micromolar range both in solution and in the gas phase. The selectivity towards BAs is more pronounced at 403 nm with the exception of histamine, which is preferably detected at 377 nm. Upon drop-coating of a layer of a mixture of 5 mol% of dye with polymethyl methacrylate on glass slides, putrescine and n-butylamine responded within 2 h to the gaseous BAs by decolorization whereas ammonia and secondary amines show no response.

A method of putrescine derivatization was established [31] using *o*-phthalaldehyde (and thioglycolic acid as a reductant) to generate red Schiff base derivatives (PUT-RD) which could be detected by the bare eye and spectrophotometically at *λ*_max_ of 490 nm. The concentration of putrescine detected in 10 fish products was varying from 11 to 190 mg/kg with an apparent recovery in the 94–106% range.

## Luminescence readout

Luminescence readout commonly is more sensitive by some orders of magnitude compared with photometry or reflectometry but formerly seemed to be complicated from an instrumental point of view and its evaluation was not easy for the layman. The requirement of an excitation light source and optical devices for selection of excitation and emission light together with blocking excitation light off the detector show that luminescence setups are more demanding and expensive than photometric and reflectometric schemes. Aside from the classical rectangular arrangement of excitation and emission light to read planar sensor membranes, fiber optic setups became very popular over the last decades. Those share the advantages that the excitation light can be brought closely even to remote samples and be read out even through transparent polymers (e.g., detection through the wall of a plastic vessel or food package with a sensor layer fixed at the inside wall). Moreover, if the excitation light is guided to an array of sensors or fed into a several optical fibers (with different sensors attached), multi-analyte sensing becomes feasible. Nevertheless, the use of simple tools like LEDs or handheld UV lamps for excitation and digital camera–based luminescence readout enabled in-field setups for less than 1000 US $, which can be operated by non-trained personnel [11]. Digital cameras and smart phones became very popular devices for luminescence detection, as well, but the demands for rejection of scattered excitation or ambient light are more demanding than in reflectometry or photometry.

A recent example uses an indicator dye (Py-1) comprising pyrylium groups [11] that responds only to primary but not to secondary and tertiary amines on dipsticks. The BA nucleophile converts the pyrylium moiety of Py-1 into the respective pyridinium salt which can visually be seen by a color change from blue (${\lambda}_{\mathrm{max}}^{\mathrm{abs}}$ = 605 nm) to red (${\lambda}_{\mathrm{max}}^{\mathrm{abs}}$ = 503 nm). Concomitantly, fluorescence appears at 602 nm with a quantum yield up to 0.5. Filter paper, indium tin oxide (ITO), and a microtiter plate were shown to be useful solid supports for quantitative analysis of the total content of BAs (TAC) [11, 25, 32] in real samples. A poly (acrylonitrile)-based hydrogel (Hypan) or cellulose acetate was chosen as polymer matrices to embed the Py-1 dye either in a sensor film or in electrospun nanofibers. Depending on whether in-field readout or quantitation in high-throughput is desired, the sensor cocktail can either be dip-coated on a filter paper to yield dipsticks or be deposited in the wells of a microtiter plate. A standard fluorescence microtiter plate reader was then used to monitor the aging of meat and cheese over time reliably and reproducibly which was confirmed by GC-MS [32]. Signal acquisition from dipsticks was performed upon excitation either in a black box with LEDs (*λ*_exc_ = 505 nm) [11] or with a UV lamp [25] (at 254 nm) and subsequent fluorescence readout with a digital camera from RGB images taken in RAW format. Extraction and calculation of the fluorescence intensity ratio by free ImageJ software deliver the concentrations of all biogenic amines in a sample no matter if meat, seafood, or cheese is investigated. Electrospun nanofibers on ITO dipsticks delivered a higher sensitivity [25] due to their high porosity and surface area. Here, the anionic CA fibers are counter-charged with respect to the BAs and additionally serve for pre-concentration. Importantly, the dipsticks additionally deliver a visual no/yes (blue→red) answer to evaluate if the BA concentrations exceed the permitted level or not.

An aminodiacetic acid–modified Nile red–Ni^2+^ complex and calcein blue complexed with Fe^2+^ ions served as luminescent probes for histamine [45] and dopamine [46]. Metal-induced luminescence quenching was switched off here upon ligand exchange with BAs. This also could be used in assays with a shortwave detection wavelength (440 nm) at micromolar concentrations of dopamine.

A commercial electrospun nanofiber mat (Tiss®-Link) was used as a support material for the direct highly selective and sensitive determination of tryptamine (LOD 6 ng/mL, LOQ 19 ng/mL) in beer [33]. Activated vinyl groups on the surface of the mat permit a fast covalent immobilization of tryptamine by means of a Michael-type reaction so to form the transduction system. The intensity of solid surface-room temperature phosphorescence (SS-RTP) is measured at 443 nm (*λ*_exc_ = 290 nm) after spotting with KI solution and total removal of oxygen from the measurement cell. The selective detection of putrescine and cadaverine based on quenching of a luminescent anthracene-Fe^3+^ chelate due to decomplexation in aqueous DMSO enabled sensing in ppb levels in both vapor phase and solution using the chelate coated on alumina or inexpensive paper strips, respectively [47].

A fiber optic enzymatic biosensor uses the oxidation of amines under oxygen consumption for determination of putrescine, cadaverine, spermidine, and histamine [34]. Diamine oxidase (DAO) was immobilized on two types of magnetic particles based either on magnetite covered with chitosan or on commercial SEPABEADS® EC-HA 403 with a ferrofluid. Embedding of the DAO magnetic particles and the Ru-bathophenanthroline complex into an inorganic–organic hybrid polymer ORMOCER® KSK 1238 yielded a cocktail that was deposited on a PMMA lens. The lens was fixed at the tip of an optical fiber in a gas-tight steel tube. The increase of the fluorescence lifetime (and decrease of oxygen quenching) of the ruthenium complex was proportional to the concentration the BAs at micromolar levels. Lifetime measurements need slightly more complex instrumentation but have the benefit of being independent of fluorophore aging, leaching, and aging of the excitation light source. Even though, the instrumentation employed is small enough to make the sensor system amenable to on-site analyses.

New classes of materials also find their way into food sensing with a lanthanide metal–organic framework (EuMOF) comprising an organic dye (methyl red, MR) [48]. This sensing material was used for determination of histamine using an advanced analytical device based on a one-to-two logic gate. After exposition to histamine vapor, the fluorescence intensity of Eu^3+^ at 613 nm decreased (3-fold) and the maximum emission of MR increased (44-fold) and a color transition under a UV lamp from red to blue occurred with a response time of 25 min.

A novel sensor chip based on a graphene oxide (GO) aerogel and photonic crystals (PCs) was fabricated. Its setup mimics the human nose with olfactory cilia and olfactory glomeruli for analyte binding and fluorescence signal processing, respectively [49]. It has the potential to discriminate ten biogenic amines and seven drug amines. Three fluorophores (acridine orange, rhodamine 6G, and rhodamine B) were chosen for multiple fluorescent sensing of BAs with various structures. For this purpose, PCs were prepared from poly (styrene/methyl methacrylate/acrylic acid) latex particles with three different diameters, which matched the emissions of 3 fluorescence dyes. The competitive interactions of fluorophores and amines to GO modulate the fluorescence emission. The analyte discrimination is achieved chemometrically by additional linear discriminant analysis and hierarchical clustering analysis.

Histamine fluorescence sensing was also established using CdSe/ZnS quantum dots which were modified by a 1-vinyl-3-butyl-imidazolium hexafluorophosphate ionic liquid (QDs@IL@MIP) [50]. The surface of the QDs was covered with poly-methacrylic acid into which histamine was embedded as molecular imprint. The fluorescence of QDs@IL@MIP at 605 nm (*λ*_exc_ = 400 nm) was enhanced by histamine in a concentration range of 0.449–2.249 mM with a LOD of 0.11 mM.

## Chemiluminescence readout

Chemiluminescence (CL) can be detected after a product of a chemical reaction is generated in an electronically excited state and returns to its ground state under emission of light. The underlying kinetic of the reaction imposes a transient signal, which often is amplified by coupling with an enzymatic reaction and/or an enhancer molecule for analytical purposes. Most importantly, as no excitation light is required, much less background signal and scatter from the matrix of real samples can be expected to occur when using CL detection as compared with reflectometry, photometry, or luminescence detection. This yields very low detection limits in many cases provided that no cross-reactions of enhancer and analyte molecule occur. Further, the absence of an excitation source makes instrumentation for these methods considerably simpler because just a light-tight detection cell, sample holder, light-collecting lens, and a sensitive photodiode are required. This way, CL detection is even more simple than reflectometry or photometry, and hence, CL sensors have the potential for miniaturization and on-site analysis. These advantages hold true, if CL is coupled, e.g., with enzymatic signal enhancement and luminol, and also imposes restrictions in the selection of analytes via the choice of the enzyme. Further, there are much less CL reactions (and with it potential analytes a sensor can respond to) than other chemosensors which limits the use of this detection method.

Enzymatic biosensor membranes based on hydroxyethylcellulose [18] provide an indirect determination of BAs via the H_2_O_2_ formed from putrescine with under the action of putrescine oxidase or diamine oxidase, respectively. Here, the Co(II)-catalyzed reaction with luminol produces chemiluminescence that is proportional to both the concentration of the hydrogen peroxide as well as the concentration of putrescine. Such biosensors were used for determination of BAs in meat and fish samples (LOD at 1 mg/L level) and showed comparable results with HPLC with precolumn derivatization. A potential in-field use can be envisioned if minimized instrumentation would be used.

Mg–Al–CO_3_ double-layered hydroxide (LDH) nanosheets show strong blue photoluminescence (*λ*_exc_ = 365 nm) which is decreased by BAs through a displacement of the O–H⋯O bonds by O–H⋯N between BAs and sheets. The decreased catalytic effect of these LDHs on the chemiluminescence of the bis(2,4,6-trichlorophenyl) oxalate (TCPO)–H_2_O_2_ system successfully enabled histamine determination in spoiled fish and pork meat samples [35].

## Total internal reflection ellipsometry

Thin hybrid films consisting of copper phthalocyanines on single-walled carbon nanotubes (SWCNTs) were applied for sensing amine vapors [21] by total internal reflection ellipsometry. Those films were produced by spin-casting the solutions onto gold-coated slides and onto silicon substrates. Ellipsometry determines the change of the polarization state of light upon its reflection at a sample when irradiated with linearly or circularly polarized light. In spectroscopic ellipsometry, the parameters *Ψ* and *Δ* are monitored in the respective wavelength range (400–1000 nm in [21]) with an ellipsometer. The tan *Ψ* is the modulus of the complex ratio of the reflection coefficients. In the present publication, shifts of *Δ* are induced on the hybrid films within 1–2-min response time by adsorption of amine vapors (methylamine, dimethylamine, trimethylamine). Those phase shifts are due to the phase shift between the p- and s-components of polarized light and increase with the concentration of the BAs. Methylamine was detected with an LOD of 3.6 ppm while the steric hindrance of diethylamine and trimethylamine reduces their response. As ellipsometry more and more finds its way into sensing, there is the hope that the miniaturization of the bulky instrumentation and the simplification of the demanding data evaluation of this detection method will open a window for its in-field use for food analysis.

## Surface-enhanced Raman spectroscopy

SERS uses the massive increase of a Raman signal which can occur once an analyte interacts with a surface that has a nanostructured metallic surface. Here, an electromagnetic and a chemical fraction contribute to the overall signal enhancement. The chemical enhancement originates from the greater polarizability of the molecule that is adsorbed onto the SERS substrate. Additionally, the incident light excites localized surface plasmons on the metallic surface which create an electromagnetic field that can be enhanced strongly (the electromagnetic enhancement). Enhancement factors up to 10^14^ have moved detection limits of this method to the single molecule level, albeit this concentration level cannot be expected in real samples but is obtained with research-level instrumentation, only. The main limitations of SERS-based BA detection in food analysis are the relatively high price of a portable Raman spectrometer (10,000–30,000 Euro) and the requirement for noble metal nanoparticles in the sample or a nanopatterned surface for quantitation. Moreover, temperature has to be stabilized very carefully to eliminate potential interferences on the measurement. On the other hand, SERS does not require labeling of the target analytes and provides on-line sensing capability in flow cells.

Although SERS nowadays becomes more widely used for food analysis [51] with chromatographic separation techniques, the first report on its application for BAs detection in food was published in 2015 [36]. As histamine is one of the most popular BAs for monitoring of fish spoiling, this BA is also most widely used in new SERS-based methods. For example, Gao et al. [36] detected histamine in artificially spiked canned tuna meat. In order to separate the analyte from the complex matrix and to minimize the background signal, the authors performed solid-phase extraction of the analyte from tuna extract by a polyvinyl chloride film with immobilized molecularly imprinted polymers (MIP). Then, a SERS substrate (a solution of AuNPs) was used for both analyte elution and detection of histamine. Liquid-liquid extraction [37] and TLC [38, 52] were also used as sample pretreatment techniques prior to SERS-based histamine detection in fish meat. Most notably, SERS detection was performed directly on the developed TLC plate which additionally shortens analysis time. With this method, Tan et al. could not only detect aging of artificially spiked meat but also monitor fish spoiling over 48 h at room temperature. All reports allow for reliable quantification of histamine in real samples within the ranges of concentrations required for detection of the spoiling process (> 100 mg/kg) (see Table 2). A chemometric analysis (e.g., principal component analysis) was also widely used in order to improve the analysis performance [36, 37, 52]. The comparison of results obtained by SERS and HPLC [38] shows that the TLC-SERS protocol with simple partial least square regression analysis also has acceptable precision (RSD < 10%). Additionally, the effect of TLC sample pretreatment on the reduction of the RSD is significantly larger than that of the chemometric treatment making a sample pretreatment step mandatory for reliable analysis [52]. This is in accordance with the general rule that sampling and sample pretreatment have more impact on precision of an analysis than the detection method itself, if real samples are studied. Finally, an advantage of all listed protocols [37, 38, 52] is the use of simple AuNPs solutions as SERS substrates with appropriate reliability and efficiency in the sample pretreatment process.

Besides detection of histamine (e.g., in seafood and fermented foodstuff), SERS can be successfully used for monitoring the decomposition (spoiling) of non-fermented foods, as well. For example, Wu et al. [53] proposed MIP-coated silver nanoparticles (AgNPs) for direct SERS detection of histamine in solution down to 0.1 mg/L. The assay for histamine detection in spiked samples showed an appropriate accuracy with apparent recovery in the ranges 85–117% (canned tuna) and 93–108% (red and rice wines).

Besides histamine, there are also some reports on detection of tyramine. Wang et al. [39] combined SERS with high-performance TLC for tyramine detection in cheese. The authors successfully detected tyramine at 30–80 mg/kg in cheese (not spiked) with an apparent recovery within the 84–108% range.

As a conclusion, SERS expects a bright future as an on-site sensing method in food control if the prices for instruments will decrease with an increasing number of purchased devices and if further miniaturized instruments will be available. It has all the merits to become a viable on-site detection method provided that suitable selectivity can be obtained with a simple sample pretreatment.

## Conclusion and outlook

This review shows that a wide variety of optical sensors and methods for BA quantification in food exist that have the potential for future commercialization due to simplification of the sampling and detection schemes and proven applicability to real food samples. Presumably, the most widely used detection methods will comprise either photometry, reflectance, or chemiluminescence using cell phone analysis due to the simplicity of the required instrumentation. However, also digital photographic sensor schemes seem promising because they can acquire photometric, reflectometric, or even fluorescence responses of a chemosensor with good reproducibility in-field at low costs. A fully automated software evaluation of the optical sensors is not available, yet although highly essential for on-site use with less educated staff, which should comprise chemometric data treatment because it has proven to enhance selectivity and reliability of food sensors. Chemometric data treatment is a further step companies could implement to bring food sensing schemes closer to home users and which could be applied to dipstick sensors provided that a simple sample preparation exists. SERS expects a bright future as an on-site sensing method in food control, if the prices for instruments and nanopatterned detection cells will decrease with an increasing number of purchased devices and if further miniaturized instruments will be available. It has all the merits to become a viable on-site detection method provided that suitable selectivity can be obtained with a simple sample pretreatment.

Optical food sensing is also a chance for scientists working with microfluidics to get involved into a new inspiring field of sensor science because miniaturized microfluidic systems could also include automated sample pretreatment steps and combine them with detection inside a micro total analysis system. Hence, there are many access points and techniques to create new optical food sensor schemes and to adapt them to the wealth of analytes in real samples that may be analyzed by home users and the food industry and within food chains all over the world.
